# Phillyrin ameliorates DSS-induced colitis in mice via modulating the gut microbiota and inhibiting the NF-κB/MLCK pathway

**DOI:** 10.1128/spectrum.02006-24

**Published:** 2024-12-19

**Authors:** Tong Li, Guiqiu Hu, Shoupeng Fu, Di Qin, Zheyu Song

**Affiliations:** 1 College of Veterinary Medicine, Jilin University, changchun, China; 2 College of Animal Science and Technology, Jilin University, changchun, China; 3 Department of Gastrointestinal, Colorectal and Anal Surgery, China-Japan Union Hospital of Jilin University, changchun, China; University of the Pacific, Arthur A. Dugoni School of Dentistry, San Francisco, California, USA

**Keywords:** phillyrin, DSS, UC, gut microbiota, intestinal barrier

## Abstract

Phillyrin (PHY), also known as forsythin, is an active constituent isolated from the fruit of *Forsythia suspensa* (Thunb.) Vahl (Oleaceae). It exhibits anti-inflammatory, anti-viral, and antioxidant properties. However, the precise impact of PHY on colitis induced by dextran sodium sulfate (DSS) and its mechanism remain elusive. The present investigation revealed that PHY (12.5, 25.0, and 50.0 mg/kg) exhibited significant therapeutic efficacy in protecting mice against DSS-induced colitis. This effect was manifested as reduced weight loss, a shortened colon, increased secretion of inflammatory factors, increased intestinal permeability, and an enhanced disease activity index in mice with ulcerative colitis (UC). Molecular investigations have determined that PHY mitigates the nuclear translocation of nuclear factor kappa B, thereby downregulating myosin light-chain kinase-driven myosin light-chain phosphorylation. This mechanism results in the preservation of the integrity of the intestinal barrier. The outcomes of 16S rRNA sequencing suggest that PHY (50 mg/kg) augmented the relative abundance of certain probiotic strains, including *Lactobacillaceae* and *Lachnospiraceae*. Additionally, PHY supplementation elevated the short-chain fatty acid contents within the intestinal contents of mice with UC. In conclusion, pre-treatment with PHY may ameliorate the DSS-induced UC in mice by lowering the expression of inflammatory factors, protecting intestinal barrier function, and enhancing the structure of the intestinal flora.

**IMPORTANCE:**

The protective effect of phillyrin on DSS-induced colitis was explained for the first time, and the anti-inflammatory effect of phillyrin was demonstrated by fecal microbiota transplantation experiments mainly through intestinal flora.

## INTRODUCTION

Inflammatory bowel disease (IBD) constitutes a non-specific, chronic gastrointestinal inflammatory disorder encompassing Crohn’s disease and ulcerative colitis (UC) (1, 2). While the exact mechanism of UC remains uncertain, it is hypothesized to involve genetic predispositions, environmental influences, immune dysregulation, and other contributing factors (3). The primary clinical manifestations of UC comprise abdominal pain, diarrhea, mucous, pus, and blood in the stool (1). Moreover, pharmacological interventions exhibit considerable constraints, with prolonged usage of certain medications leading to irreversible side effects (4). Plant metabolites not only have anti-inflammatory, anti-bacterial, and other effects but also have fewer side effects (5, 6, 7). Research on the use of natural plant metabolites for the prevention and treatment of inflammatory bowel disease has garnered significant interest (8, 9).

Consequently, investigations into utilizing natural compounds to prevent and treat IBD have garnered considerable attention (10). Research indicates that individuals with IBD exhibit a disrupted balance of intestinal microorganisms. This imbalance is characterized by reduced beneficial bacteria and an elevation of harmful strains. Consequently, it leads to intestinal damage in comparison to their healthy counterparts (11, 12). The imbalance in intestinal flora disrupts the permeability of the intestinal mucosa. This disruption enables pro-inflammatory substances, such as antigens and endotoxins, to traverse the intestinal mucosa. Consequently, it triggers an inflammatory response, causing further damage to the intestine (13). Therefore, resolving the imbalance of intestinal flora and enhancing the intestinal barrier may be an effective treatment for IBD.

Phillyrin (PHY) is an endophytic fungal metabolite isolated most notably for *Forsythia suspensa* (Thunb.) Vahl (Oleaceae). It exhibits anti-oxidation (14), anti-obesity (15), and anti-inflammatory properties. For example, PHY alleviates Lipopolysaccharides (LPS)-induced lung inflammation in mice by inhibiting mitogen-activated protein kinase (MAPK) and nuclear factor kappa B (NF-κB) activation (16). However, the mechanism by which PHY modulates intestinal flora and alleviates inflammation in UC has yet to be elucidated. Therefore, the present investigation explored the preventive effect of PHY on acute UC and its underlying mechanism.

## MATERIALS AND METHODS

### Animal experiments

PHY (98%) was purchased from Shanghai Yuanye Biotechnology Co., Ltd. (Shanghai, China). C57BL/6 mice (6–8 weeks old, *n* = 6) were purchased from Liaoning Changsheng Biotechnology Co., Ltd. The mice were allowed to drink water freely for 1 week. Based on previous studies, PHY concentrations were selected (17, 18). The animals had unrestricted access to water for a duration of 1 week. Afterward, they were randomly classified into six groups, each comprising six mice: a control group, a PHY (12.5 mg/kg) group, a dextran sodium sulfate (DSS) (2.5%) group, and a DSS + PHY (12.5, 25.0, and 50.0 mg/kg) group. The mice were given PHY from the first day of the study and continued receiving it for 15 days. DSS (2.5% wt/vol; MP Biomedicals, Santa Ana, CA, USA) was introduced into their drinking water from days 15 to 23 to induce a murine model of colitis.

### Fecal microbiota transplantation

#### Donor mice

The experimental design is depicted further (see Fig. 11A). Initially, male C57BL/6 mice aged 6–8 weeks (*n* = 6) were classified into control, DSS, and PHY treatment groups. The mice were given PHY from the first day of the study and continued receiving it for 16 days. DSS was introduced into their drinking water from days 16 to 23 to induce a murine model of colitis. Fresh feces were collected daily from each mouse at a dosage of 50 mg and then mixed with sterile saline. The mixture was subjected to centrifugation at 1,000 × *g* for 5 min to collect the supernatant. Following this, the supernatant was administered to each recipient mouse by gavage.

#### Recipient mice

Male C57BL/6 mice aged 6–8 weeks (*n* = 6) were classified into the fecal microbiota transplantation (FMT)-control, FMT-DSS, and FMT-PHY + DSS treatment groups. Initially, the mice received 0.2 mL of a fresh antibiotic cocktail (containing vancomycin 0.5 g/L, metronidazole 1.0 g/L, colistin 1.0 g/L, and neomycin 0.5 g/L) for 14 days. After 14 days, fecal supernatant from the donor mice was then transplanted into the recipient mice for 16 days. After 16 days of transplantation, the mice were treated with distilled water containing 2.5% (wt/vol) DSS for 1 week. Subsequently, tissue samples were obtained for analysis after euthanizing the mice.

### Disease activity index score

The disease activity index (DAI) score assessed the clinical progression of colitis throughout the trial. This index encompasses a composite score derived from weight, fecal consistency, and bleeding parameters. The scoring criteria utilized in this investigation were adapted from previous reports (19). The scoring criteria are specified as follows: weight loss (4, 9%–12%; 3, 6%–9%; 2, 3%–6%; 1, 1%–3%; and 0, none); rectal bleeding (4, large bleeding; 1–3, partial bleeding; and 0, normal); and stool consistency (4, watery diarrhea; 1–3, sparse stool; and 0, normal).

#### Histological evaluation

After washing with pre-cooled phosphate-buffered saline (PBS), the distal colon was immediately fixed in a 4% paraformaldehyde solution and maintained for 24 h. Subsequently, the colon was fixed in dehydrated paraffin and sliced into 5-mm sections utilizing a tissue microtome. Hematoxylin and eosin staining (H&E) was utilized to evaluate the alterations in colon histomorphology. As per earlier studies (20), the histological evaluation of colonic sections involved the examination of four parameters: crypt structure, infiltration of inflammatory cells, presence of goblet cells, and formation of crypt abscesses. The images were taken with a microscope (Olympus BX41, Shanghai Puhe Optoelectronic Technology Co., LTD, Shanghai, China) with at least three fields of view per set.

### Determination of pro-inflammatory cytokines (tumor necrosis factor-alpha, interleukin-1β, and interleukin-6)

The colon tissue of mice was extracted, and an adequate amount of PBS solution was added for grinding. Subsequently, the mixture was centrifuged for 10 min at 3,000 rpm, and the supernatant was shifted into a new eppendorf (EP) tube. Inflmmatory cytokines were detected per the enzyme-linked immunosorbent assay instructions (BioLegend, San Diego, CA, USA).

### Measuring myeloperoxidase activity in colon tissue

Tissue samples of the colon were obtained, weighed, and homogenized. Upon homogenization, the mixture underwent centrifugation at 12,000 × *g* for 20 min. The obtained supernatant was utilized to assess myeloperoxidase (MPO) activity. Additionally, 75 µL of the supernatant was dispensed into a 96-well plate and permitted to react with resorcinol (6 mM, 180 µL) and H_2_O_2_ (3%, 2.5 µL) for 3–5 min. Subsequently, the MPO activities in the samples were measured at OD450 utilizing an enzyme-linked instrument.

### Detection of oxidative stress index

Colonic tissue samples were lysed with radio immunoprecipitation assay (RIPA) lysis buffer, and the cell-free supernatant (CFS) was acquired. The content of malonaldehyde (MDA), glutathione (GSH), superoxide dismutase (SOD), and catalase (CAT) in CFS was determined using a kit (Njjcbio, Nanjing, China) according to the manufacturer’s instructions.

### Immunofluorescence staining

The tissue underwent paraffin embedding to facilitate sample solidification for sectioning. Following dehydration, antigen retrieval, and sealing, the corresponding primary antibodies claudin-3 (1:100; C, Wuhan, China) and occludin (1:100, Proteintech) were incubated overnight. The slides were subsequently washed thrice in PBS for approximately 5 min each. After slight drying, goat anti-rabbit IgG (1:2,000; incubation for 1 h in Santa Cruz, CA, USA) was added at room temperature. Ultimately, 4′,6-diamidino-2-phenylindole was applied to stain the nuclei. The images were captured with a laser confocal microscope (Nikon Confocal Microscope C2, Nikon Precision Machinery (Shanghai) Co., LTD, Shanghai, China), with each group of at least three fields of view.

### Transmission electron microscope

At the end of the experiment, a 2 mm × 1 mm section of colon tissue was promptly submerged in 2%–3% glutaraldehyde. The temperature was maintained for 2 h, and the section was transferred to the refrigerator at 4°C. Subsequently, the fixed colonic tissue underwent three washes with PBS and was exposed to 1%–2% osmic acid. After 2–3 h of conventional embedding, ultrathin sections of 60 nm were processed and stained with lead citrate and uranium acetate. The resulting images were examined utilizing an HT-7800 transmission electron microscope (HITACHI, Tokyo, Japan).

### Western blotting

Appropriate amount of colon tissue was weighed into an EP tube and pre-cooled RIPA buffer was added, then protein was extracted using a Bicinchoninic Acid Assay kit (Thermo Scientific, China). Then, an equal amount of protein molecules (about 30 µg) was added to each well for 80 V, 20 min, at constant pressure. Electrophoresis was performed at 120 V and 40 min. The protein was transferred from gel to polyvinylidene difluoride membrane (PVDF) (Millipore, Darmstadt, Germany) by wet transfer method at constant flow for 220 mA and 75 min. After the transfer, 5% skim milk powder was added, and the table was closed at room temperature for 4 h. After closure, tris-buffered saline with Tween 20 (TBST) was washed three times for 10 min each time, and then the primary antibody was incubated in primary antibodies against occludin (1:1,000; Proteintech), myosin light-chain kinase (MLCK) (1:1,000; Abcam, Cambridge, UK), phosphorylated myosin light chain (p-MLC) (1:1,000; Abcam), myosin light-chain (MLC) (1:1,000; CST, Boston, USA), p-NF-κB (1:1,000; Abcam), NF-κB (1:1,000; Abcam), p-IκB (1:1,000; CST), IκB (1:1,000; CST), claudin-3 (1:1,000; Abcam), inducible nitric oxide synthase (iNOS) (1:1,000; Proteintech), cyclooxygenase-2 (COX-2) (1:1,000; Proteintech), and β-actin (1:1,000; Proteintech) at 4°C overnight. After the primary antibody was recovered, the unattached primary antibody was washed with TBST three times for 10 min each time. A suitable secondary antibody (1:5,000; BOSTER, Wuhan, China) labeled by Horseradish Peroxidase was selected and incubated at room temperature for 2 h, and then the unattached secondary antibody was washed with TBST three times for 10 min each time. The luminescent droplets (Applygen Inst. Biotech, Beijing, China) were placed on the PVDF membrane and exposed with the chemiluminescence imaging system, and then gray scale analysis was performed.

### Microbial analysis of colonic contents and short-chain fatty acid analysis

Fecal DNA samples underwent extraction and amplification, with subsequent sequencing of the V3−V4 variable region using the Illumina Miseq sequencing platform. The generated data were subsequently assessed utilizing the cloud-based platform ZHONGke New Life.

Short-chain fatty acid (SCFA) levels in the cecum were assessed via gas chromatography. Fresh fecal samples from mice (200 mg) were collected and mixed with 1 mL of deionized water. Following centrifugation at 12,000 rpm for 10 min, the resulting supernatant (1 mL) was treated with concentrated hydrochloric acid (100 µL). Thereafter, 5 mL of ether was introduced into the solution, ensuring thorough mixing, and allowed to extract for 20 min at room temperature. The organic phase extract was utilized for SCFA analysis after centrifuging for 10 min at 3,500 rpm. A debonded free fatty acid phase capillary column from Agilent Technologies was employed for gas chromatography.

### Statistical analysis

All data underwent statistical analysis through one-way analysis of variance to compare multiple groups. This analysis was carried out utilizing SPSS version 26.0 and GraphPad Prism version 8.0 software. Data were presented as mean ± standard deviation (mean ± SEM) and were considered statistically significant at *P* < 0.05.

## RESULTS

### PHY alleviates clinical symptoms in mice with DSS-induced colitis

**Fig 1:**
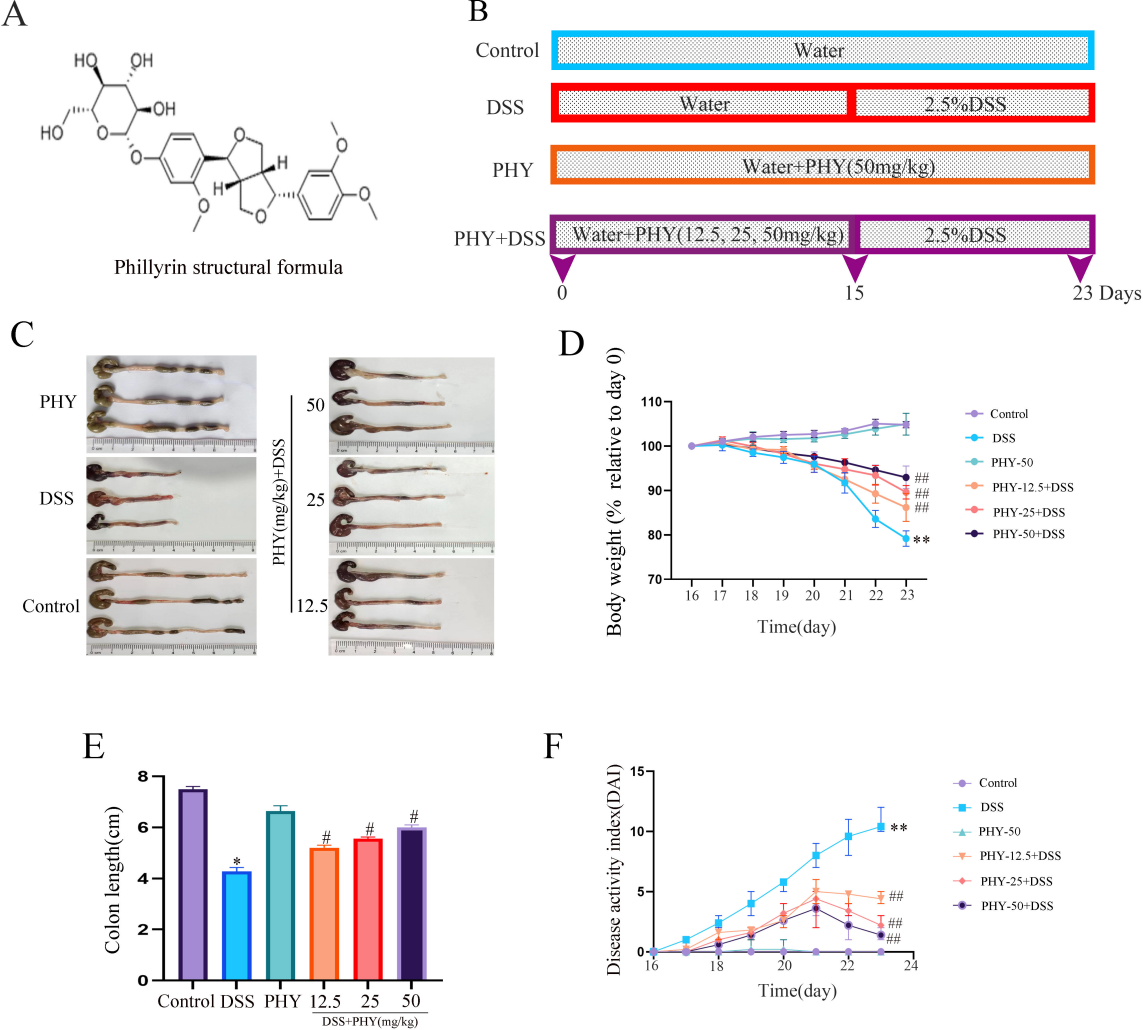
Effect of PHY on clinical symptoms of DSS-induced colitis. (**A**) Chemical structure of PHY. (**B**) Grouping of animal experiments. (**C**) Photographic documentation of colonic tissues from each experimental group of mice (*n* = 3). (**D**) Rate of weight change among mice in each group (*n* = 6). (**E**) Measurement of colon length in mice from each experimental group (*n* = 6). (**F**) DAI scores for each experimental group (*n* = 6). Data are shown as the mean ± SEM. *P < 0.05 and **P < 0.01 compared to the control group; ^#^*P* < 0.05 and ^##^*P* < 0.01 compared to the DSS group.

The chemical structure of PHY and the grouping of mice are shown in Fig. 1A and B. The grouping of mice is depicted in Fig. 1B. Clinical outcomes were evaluated by assessing weight loss and DAI. Relative to the control group, mice in the DSS group revealed a substantial weight reduction, whereas the weight loss was notably alleviated after PHY pre-protection (Fig. 1D). In UC patients and animal models, the colon underwent considerable shortening, and the formation of intestinal contents was compromised (21, 22). In comparison to the control group, the colon of the DSS group exhibited signs of swelling and congestion, accompanied by a considerable reduction in length. Following PHY pre-protection, colon length was considerably increased (Fig. 1C and E). Relative to the control group, the DAI score of the model group was markedly higher. However, treatment with PHY pre-protection significantly mitigated the DAI score (Fig. 1F). These outcomes revealed that PHY pre-protection effectively alleviates the clinical symptoms induced by DSS.

### PHY attenuated DSS-induced pathological injury in UC mice

**Fig 2:**
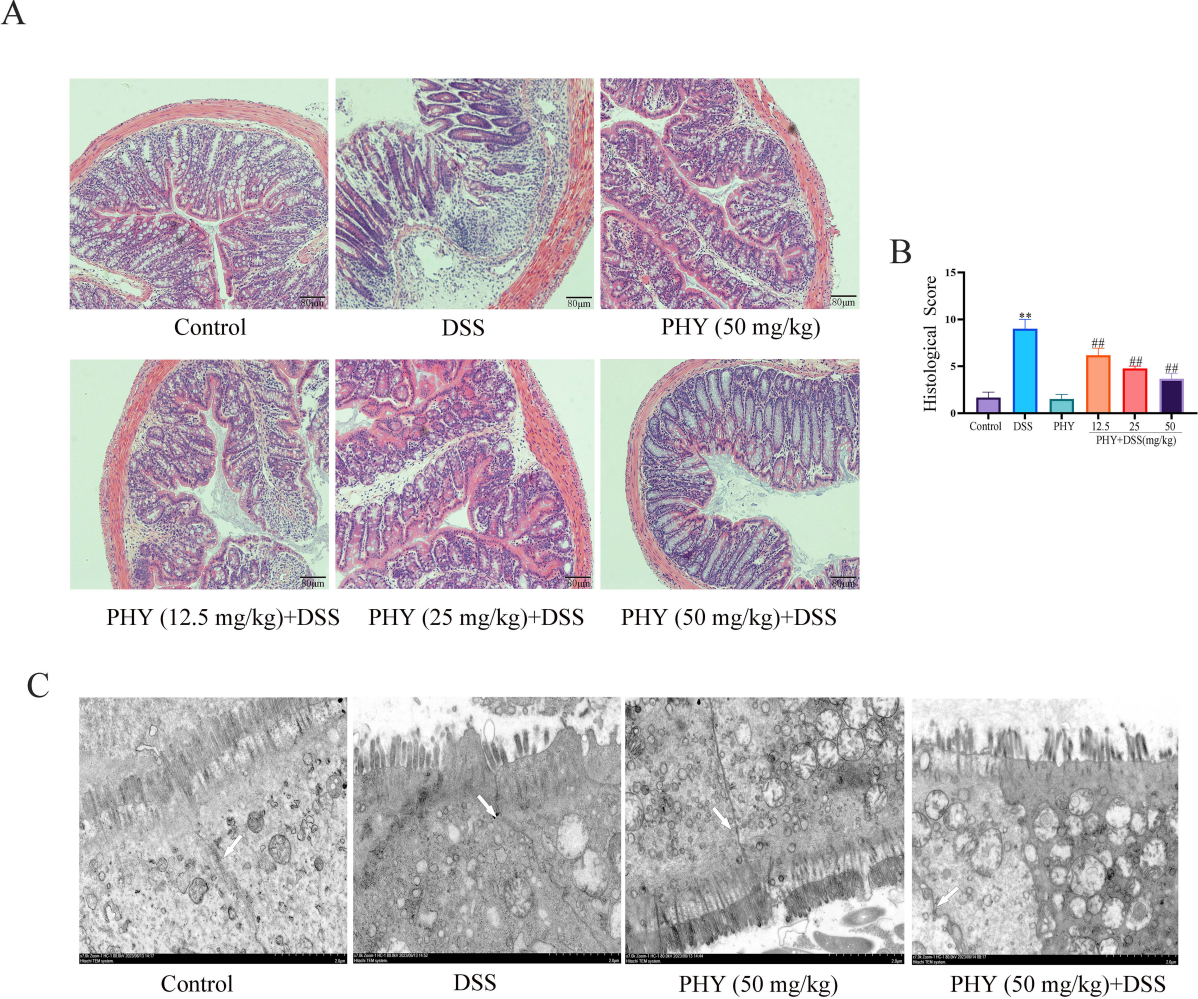
Effect of PHY on pathological damage and barrier in DSS-induced colitis. (**A**) Histopathological evaluation of tissue injury in each group utilizing hematoxylin and eosin staining. (**B**) Histological scores for individual groups; bar scale = 80 µm. (**C**) Examination of barrier structure utilizing electron microscopy; The white arrow represents the intestinal barrier (tight junction); bar scale = 2 µm. Each group consisted of six biological replicates (*n* = 6). Data are depicted as the mean ± SEM. **P < 0.01 compared to the control group; ^##^*P* < 0.01 compared to the DSS group.

The pathological lesion of mice enteritis was evaluated by H&E staining of colon sections. As depicted in Fig. 2A, the DSS group exhibited considerable inflammatory cell infiltration, along with substantial damage to intestinal crypts, in comparison to the control group. However, following PHY pre-protection, there was evident recovery from the pathological damage observed in the colon (Fig. 2B). The outcomes obtained from transmission electron microscopy revealed considerable changes in the DSS group, including enlarged cell spaces, loosened epithelial tight junctions (TJs), and swollen mitochondria. Conversely, the morphology of intestinal epithelial cells appeared relatively normal following PHY treatment (Fig. 2C). In conclusion, PHY pre-protection has demonstrated efficacy in ameliorating colon mucosal injury and substantially reducing the colon pathological score.

### PHY alleviated the colonic inflammatory response in mice with DSS-induced colitis

**Fig 3:**
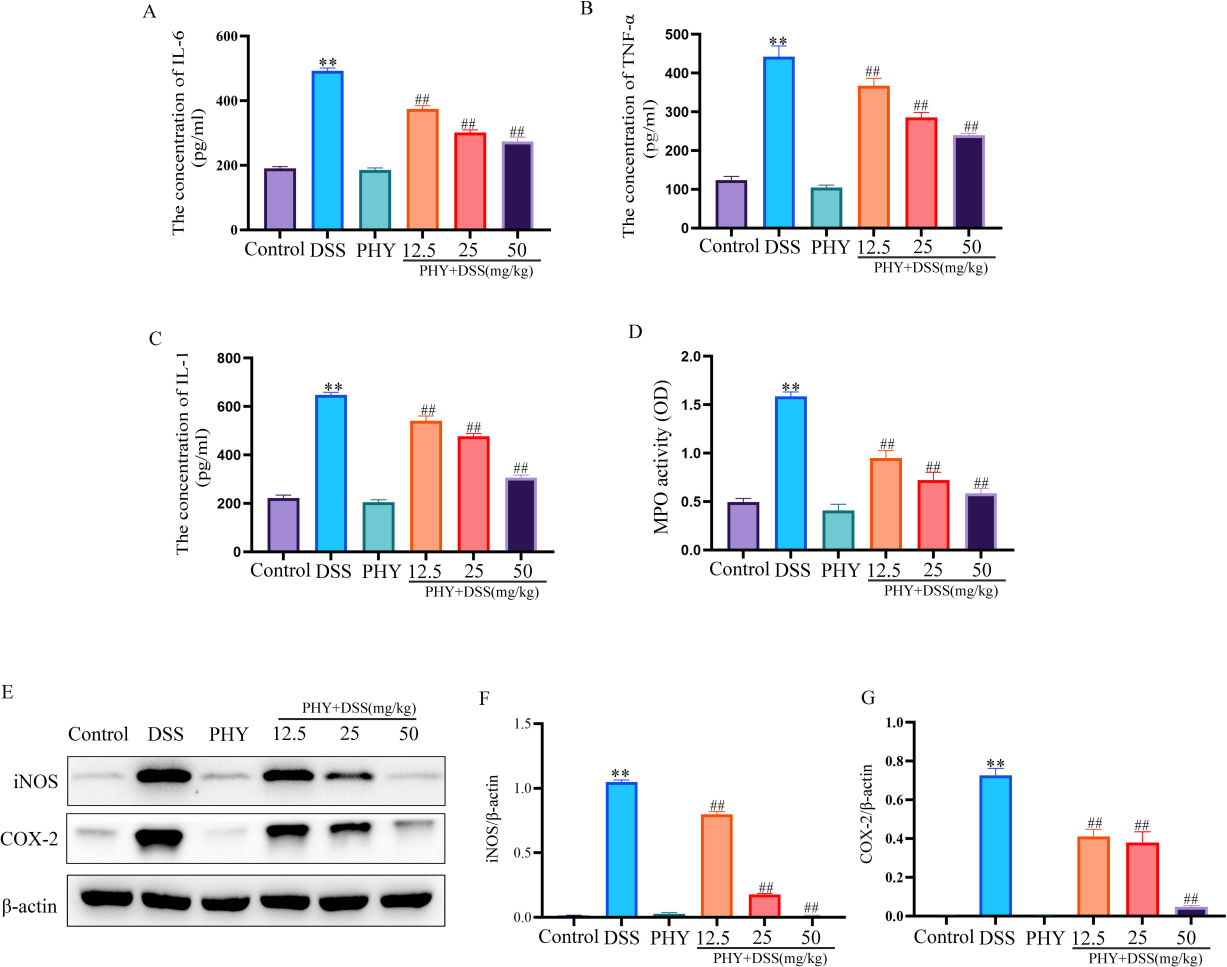
Impact of PHY on the inflammatory response of colitis induced by DSS. Evaluation of the levels of IL-6 (**A**), TNF-α (**B**), and IL-1β (**C**) by enzyme-linked immunosorbent assay. (**D**) Measurement of MPO content (*n* = 6). (**E**) Evaluation of iNOS and COX-2 protein expressions utilizing Western blotting. (**F**) Relative expressions of iNOS and (**G**) COX-2 (*n* = 3). Data are shown as the mean ± SEM. **P < 0.01 compared to the control group; ^##^*P* < 0.01 compared to the DSS group.

The outcomes indicated notably elevated tumor necrosis factor-alpha (TNF-α), interleukin (IL)-6, IL-1β, and MPO levels in the colon of the model group in comparison to the blank group. However, following PHY intervention, there was a considerable reduction in the levels of inflammatory factors and MPO in the colon (Fig. 3A through D). In comparison to the control group, the pro-inflammatory enzymes COX-2 (Fig. 3E and G) and iNOS (Fig. 3E and F) were considerably elevated in the DSS induction group. However, the expression of these pro-inflammatory enzymes in the colon was considerably reduced following the PHY intervention.

### PHY decreased oxidative stress in mice with DSS-induced colitis

**Fig 4:**
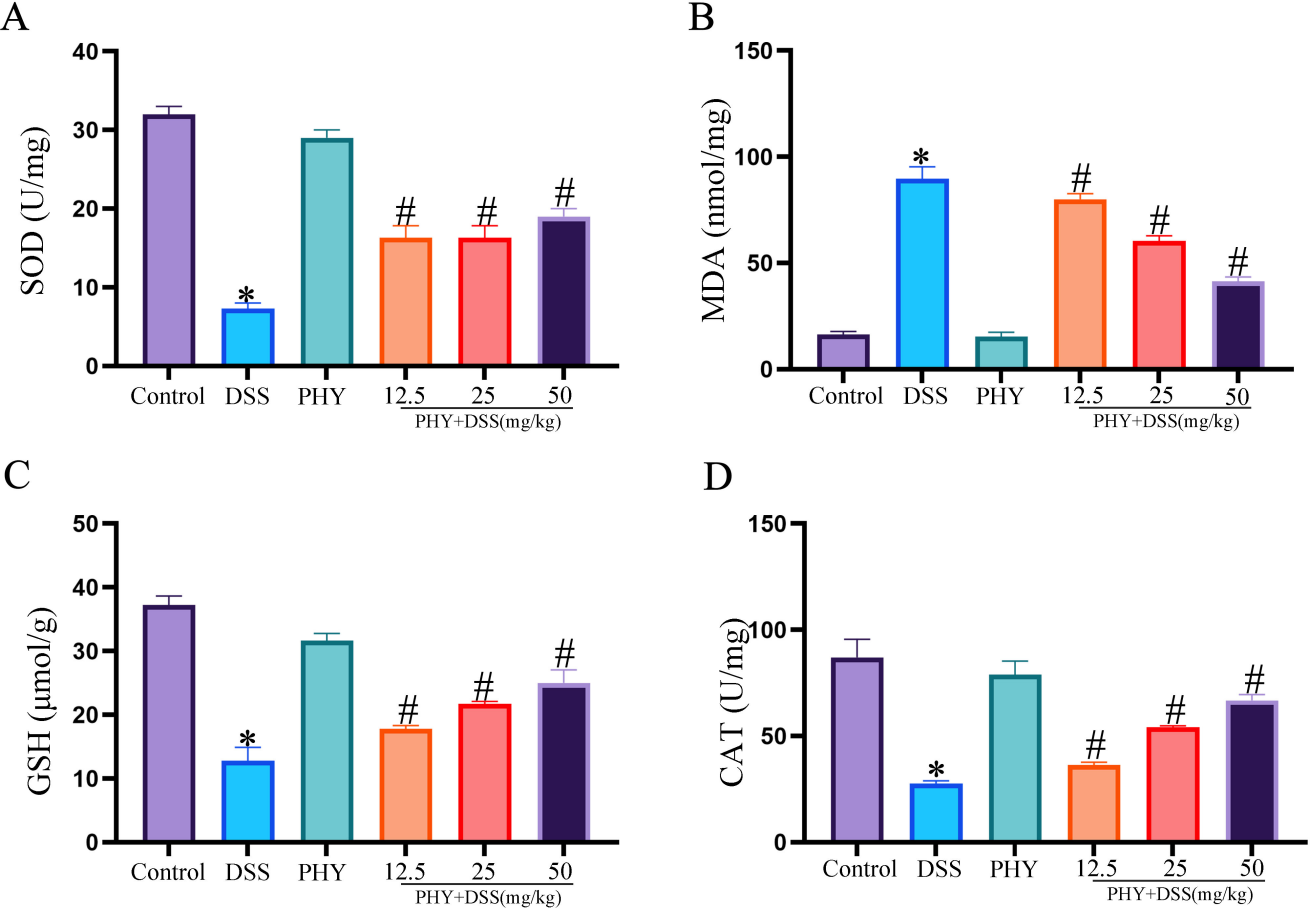
Effect of PHY on oxidative stress response in DSS-induced colitis. Detection of the contents of SOD (**A**), MDA (**B**), GSH (C), and CAT (**D**) via the kit. Each group consisted of six biological replicates (*n* = 6). Data are depicted as the mean ± SEM. *P < 0.05 compared to the control group; ^#^*P* < 0.05 compared to the DSS group.

In individuals with UC, the aggravation of inflammation can result in heightened oxidative stress within the body, leading to the generation of numerous oxygen free radicals and other oxidative byproducts such as nitric oxide and MDA. These products can cause varying degrees of damage to colon tissues. Relative to the control group, the content of MDA (Fig. 4B) in the DSS group exhibited a considerable elevation, whereas the levels of GSH (Fig. 4C), SOD (Fig. 4A), and CAT (Fig. 4D) were substantially reduced. Following the PHY intervention, there was a significant reduction in MDA activity, while SOD, GSH, and CAT activities were increased. These changes contributed to alleviating the DSS-induced oxidative stress response and repairing colon tissue damage.

### PHY improved the colonic mucosal barrier function in mice with DSS-induced colitis

**Fig 5:**
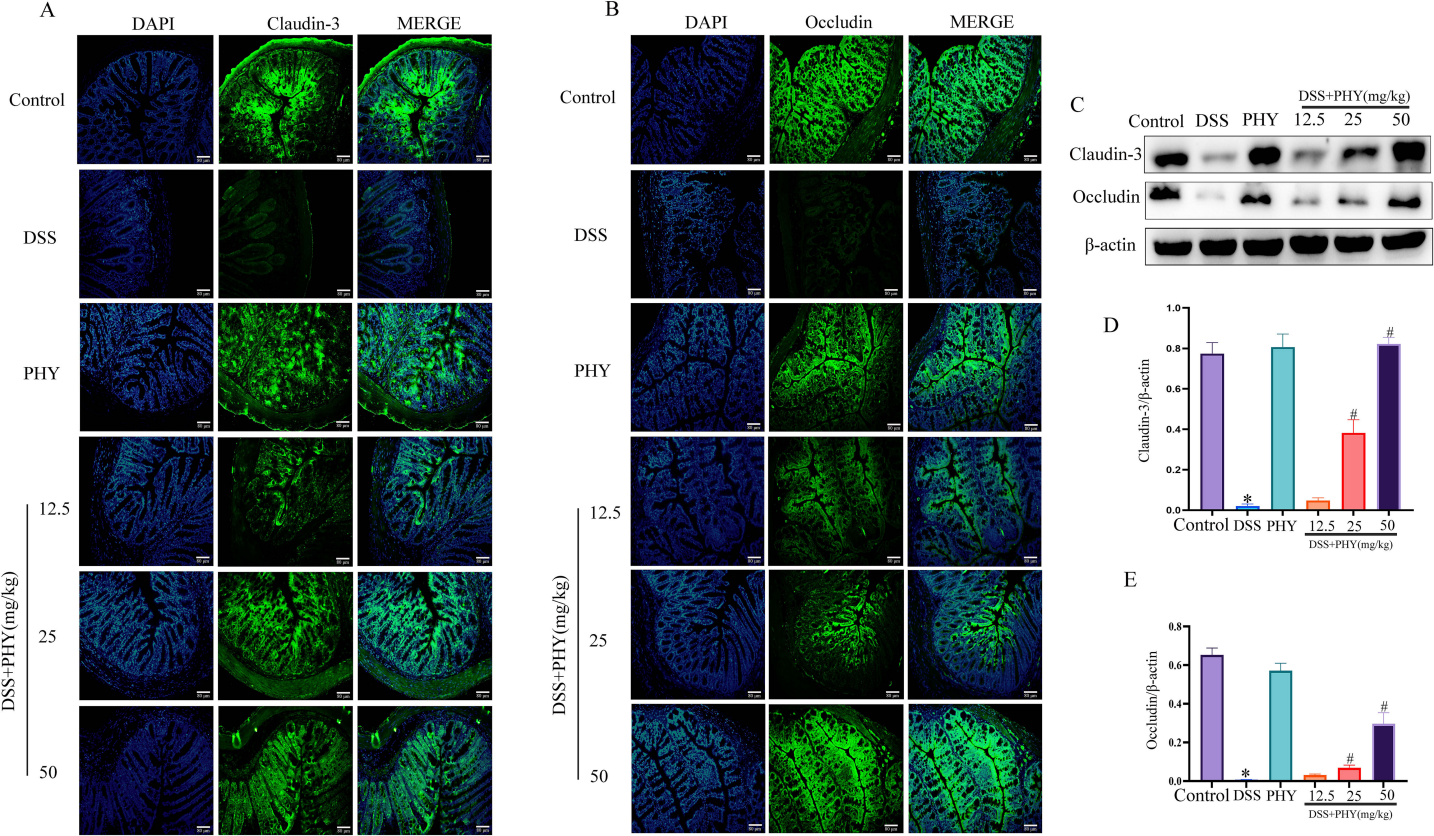
Effect of PHY on intestinal barrier in DSS-induced colitis. Immunofluorescence analysis of claudin-3 (**A**) and occludin (**B**) expression; bar scale = 80 µm. (**C**) Evaluation of claudin-3 and occludin protein expression via Western blotting analysis. (**D**) Relative protein expression of claudin-3. (**E**) Relative protein expression of occludin. Each group consisted of three biological replicates (*n* = 3). Data are depicted as the mean ± SEM. *P < 0.05 compared to the control group; ^#^*P* < 0.05 compared to the DSS group. DAPI, 4′,6-diamidino-2-phenylindole.

Immunofluorescence analysis revealed a substantial decrease in the expression of colonic TJ proteins claudin-3 (Fig. 5A) and occludin (Fig. 5B) by DSS induction. Conversely, treatment with PHY led to a considerable elevation in occludin and claudin-3 expression levels. These outcomes were consistent with the findings obtained from Western blot analysis (Fig. 5C through E). In summary, PHY can restore the colonic permeability changes induced by DSS.

### PHY repairs the intestinal barrier and reduces inflammation levels via the NF-κB/MLCK/MLC signaling pathway

**Fig 6:**
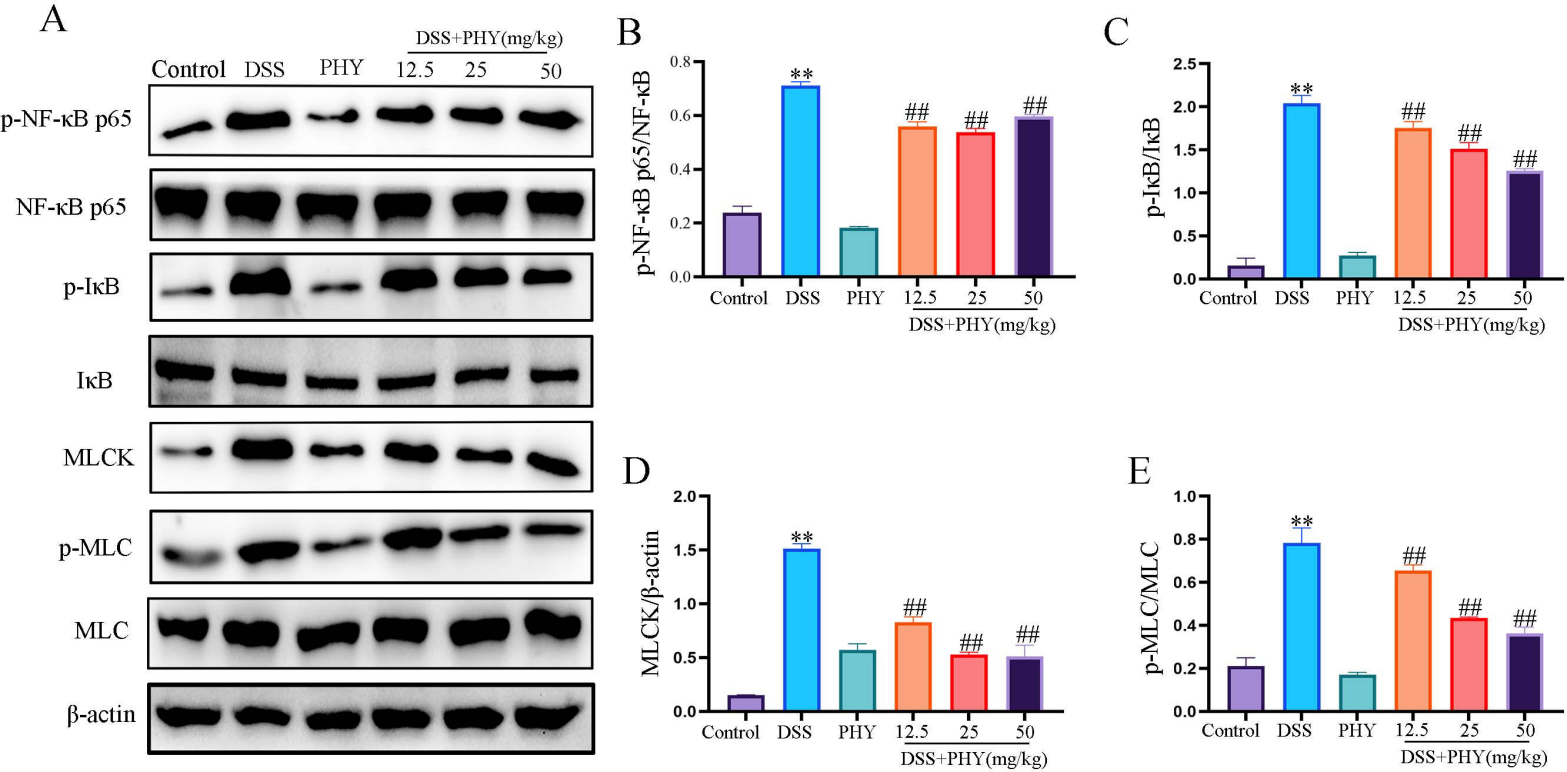
The effect of PHY on the p-NFκB/MLCK/MLC signaling pathway in DSS-induced colitis. (**A**) Detection of the protein expression of p-NF-κB, NF-κB, p-IκB, IκB, MLCK, p-MLC, and MLC by Western blotting. (**B**) Relative protein expression of p-NFκB. (**C**) Relative protein expression of p-IκB. (**D**) Relative protein expression of MLCK. (**E**) Relative protein expression of p-MLC. Each group consisted of three biological replicates (*n* = 3). Data are depicted as the mean ± SEM. **P < 0.01 compared to the control group; ^##^*P* < 0.01 compared to the DSS group.

Figure 5A illustrates that p-NF-κB p65 and p-IκB expression in the DSS group was markedly enhanced in comparison to the control group. However, the PHY group notably reversed these effects in comparison to the DSS group (Fig. 6A through C). Research has demonstrated that NF-κB can enhance MLCK transcription, increasing MLC phosphorylation and decreasing TJ protein expression (23). Furthermore, the MLCK and p-MLC protein expression in DSS was enhanced in comparison to the control group. Nevertheless, in the PHY pre-protected group, the expression of MLCK (Fig. 6A and D) and p-MLC (Fig. 6A and E) proteins was lower in comparison to the DSS group. These outcomes revealed that PHY could restore the intestinal barrier by regulating the NF-κB /MLCK/MLC signaling pathway.

### PHY increases short-chain fatty acids in mice with DSS-induced colitis

**Fig 7:**
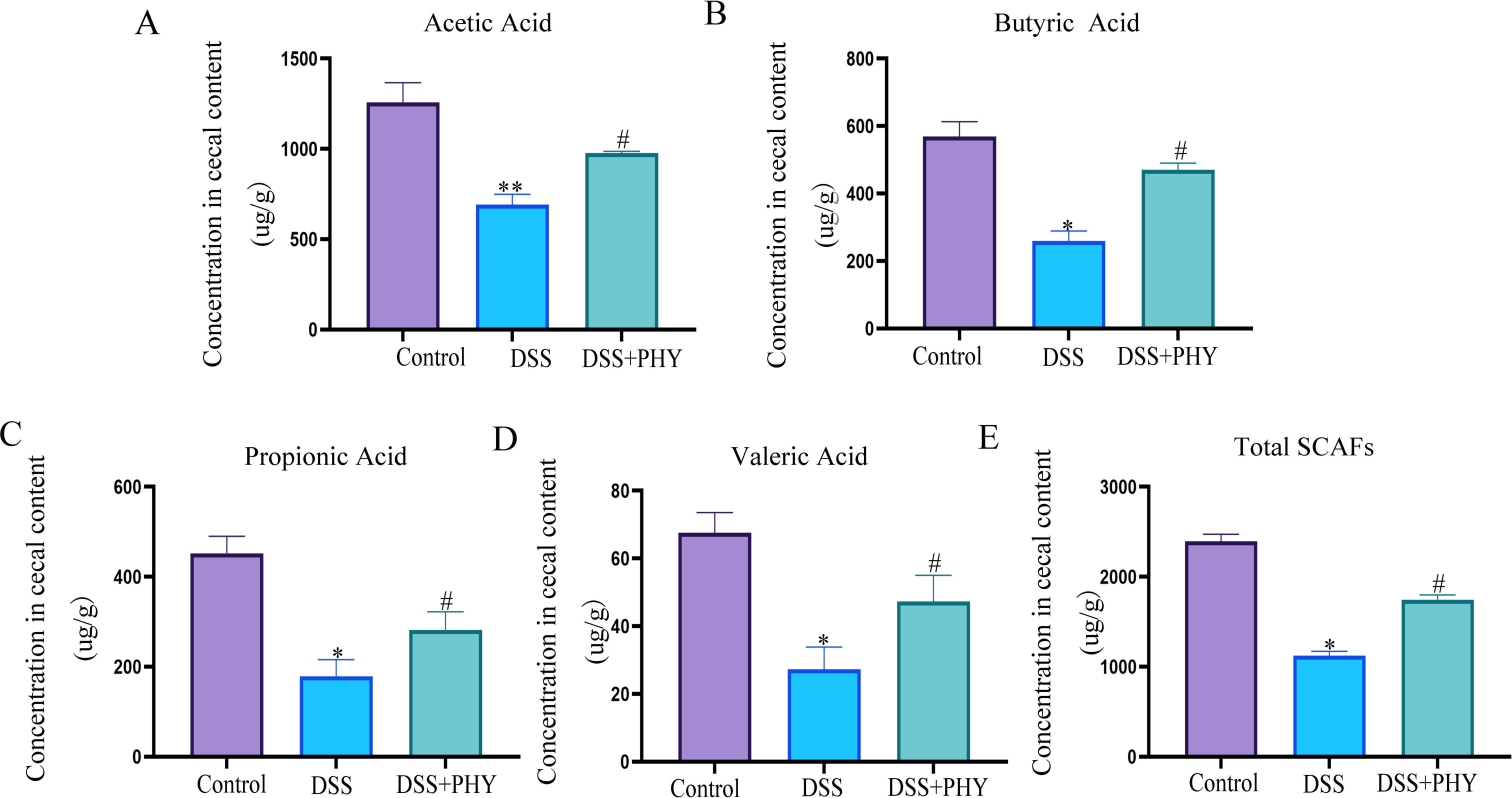
Effect of PHY on the SCFAs of DSS-induced colitis. Assessment of (**A**) acetic acid, (**B**) propionic acid, (**C**) butyric acid, (**D**) valeric acid, and (**E**) total fatty acids by liquid chromatography-mass spectrometry. PHY concentration is 50 mg/kg. Each group consisted of five biological replicates (*n* = 5). Data are depicted as the mean ± SEM. *P < 0.05 compared to the control group; ^#^*P* < 0.05 compared to the DSS group.

Previous research has revealed that SCFAs have protective, immunomodulatory, anti-inflammatory, and anti-tumor effects on the function of the intestinal barrier. The contents of SCFAs, comprising butyric acid, propionic acid, and acetic acid, in the gut of mice were assessed by gas chromatography-mass spectrometry. As depicted in the figure, in comparison to the control group, acetic acid (Fig. 7A), propionic acid (Fig. 7C), valeric acid (Fig. 7D), butyrate acid (Fig. 7B), and total SCFAs (Fig. 7E) in the gut of the DSS group were considerably reduced. However, following PHY treatment, SCFAs in the cecum of mice elevated.

### PHY regulates the composition of intestinal flora

**Fig 8:**
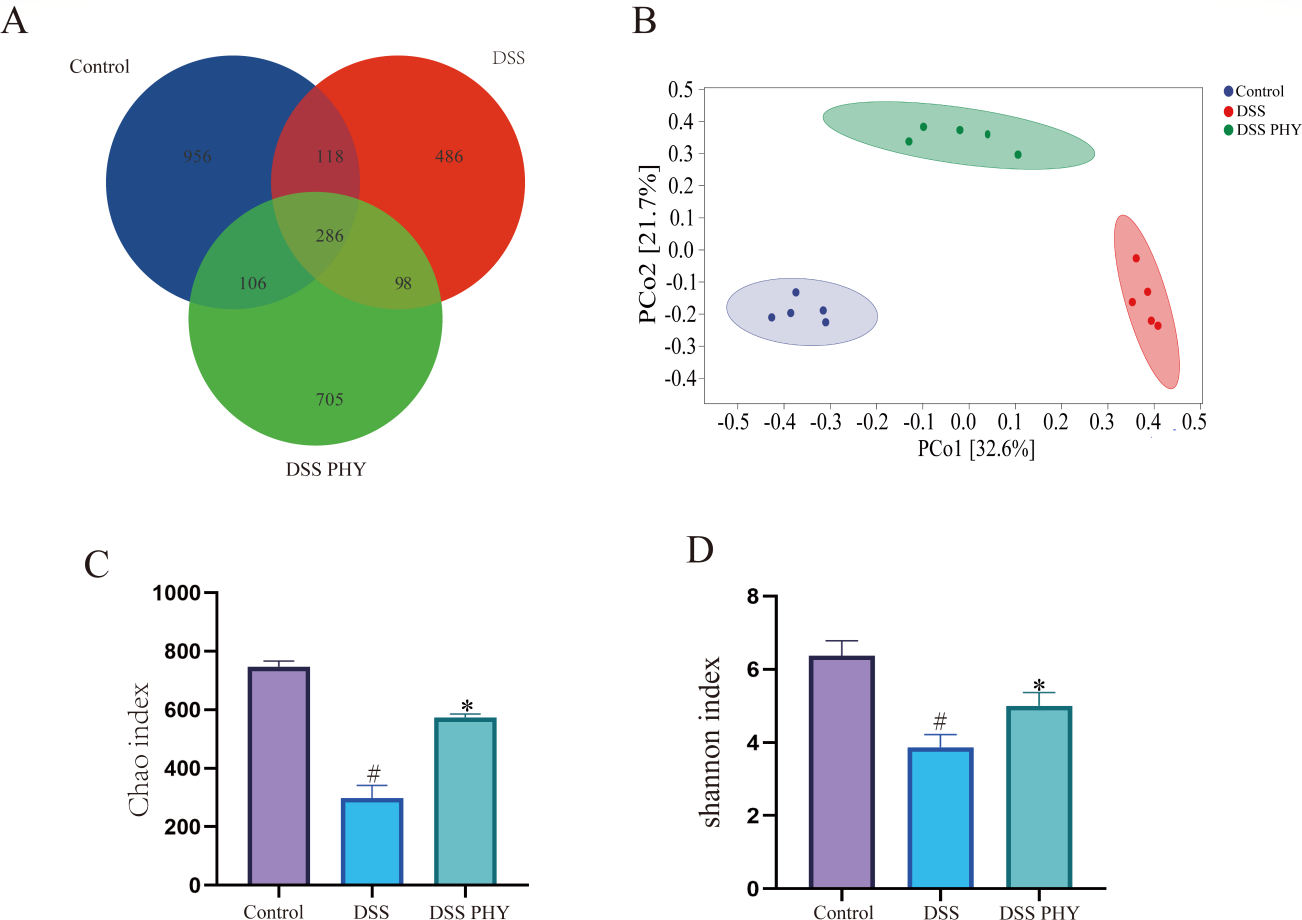
Effect of PHY on the microbiota diversity of DSS-induced colitis. (**A**) Venn diagram depicting the overlapping OTUs of intestinal flora in the control, DSS, and PHY + DSS (50 mg/kg) groups. (**B**) Principal component analysis. (**C**) Chao index. (**D**) Shannon index. PHY concentration is 50 mg/kg. Each group consisted of five biological replicates (*n* = 5). Data are depicted as the mean ± SEM. ^#^*P* < 0.05 compared to the control group; *P < 0.05 compared to the DSS group.

Subsequently, the investigation focused on whether PHY regulates the gut microbiota in mice with DSS-induced colitis. To explore this, 16S rDNA sequencing was conducted, revealing 286 operational taxonomic unit (OTUs) shared across all experimental groups, as depicted in Venn diagrams. Furthermore, the distribution of OTUs specific to the control, DSS, and PHY + DSS groups was determined to be 956, 486, and 705, respectively. Principal component analysis outcomes demonstrated significant differences among the control group, DSS group, and PHY + DSS group (Fig. 8B). Additionally, relative to the control group, the Chao1 index (Fig. 8C) and Shannon index (Fig. 8D) in the DSS group were considerably reduced, whereas these indices were considerably elevated after PHY treatment. These outcomes suggest differences in gut microbiota between different treatment groups.

**Fig 9:**
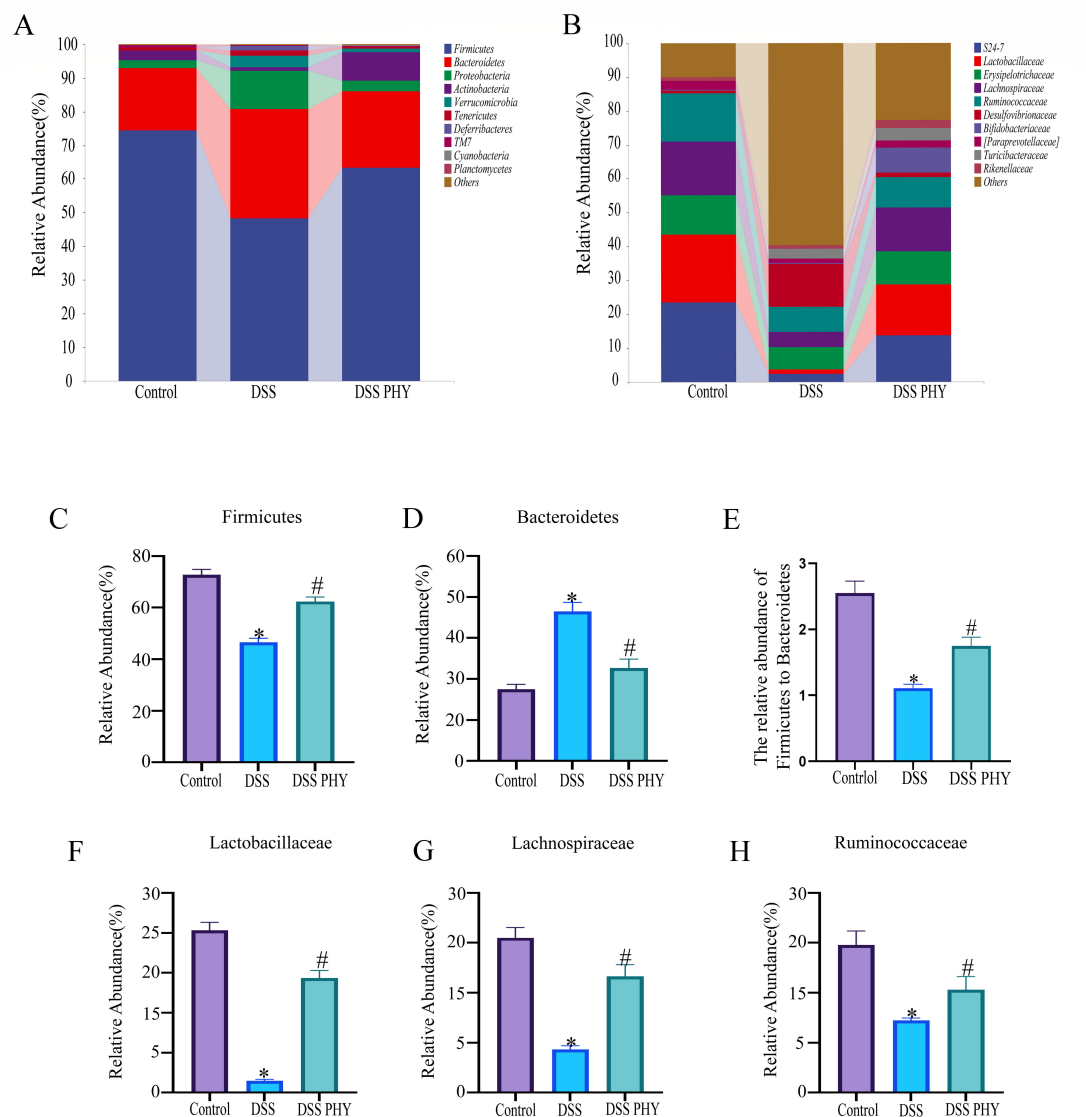
Effect of PHY on the flora abundance of DSS-induced colitis. (**A**) Histogram of relative abundance of each group at the phylum level. (**B**) Histogram of relative abundance of flora at the family level in control, DSS, and PHY-DSS (50 mg/kg) groups. (**C**) The relative abundance of *Bacteroidetes*. (**D**) The relative abundance of *Firmicutes*. (**E**) The relative abundance of *Firmicutes*/*Bacteroidetes*. (**F**) The relative abundance of *Lactobacillaceae*. (**G**) The relative abundance of *Lachnospiraceae*. (**H**) The relative abundance of *Ruminococcaceae*. Each group consisted of five biological replicates (*n* = 5). Data are depicted as the mean ± SEM. *P < 0.05 compared to the control group; ^#^*P* < 0.05 compared to the DSS group.

Variations in gut microbiota composition were evaluated at the phylum and family levels to delineate the specific composition of microbial communities in the gut. The histogram at the phylum level indicated that DSS induction resulted in an elevation in the relative abundance of *Bacteroidetes* (Fig. 9A and C) and a reduction in the relative abundance of *Firmicutes* (Fig. 9A and D). PHY pre-protection reversed these trends. Additionally, the *Firmicutes* and *Bacteroidetes* ratio in DSS-induced mice was notably elevated after PHY treatment (Fig. 9A and E). At the family level, histograms revealed that DSS induction reduced the abundance of *Lactobacillaceae* (Fig. 9B and F), *Lachnospiraceae* (Fig. 9B and G), and *Ruminococcaceae* (Fig. 9B and H) relative to the control group. However, PHY pre-protection reversed these trends.

**Fig 10:**
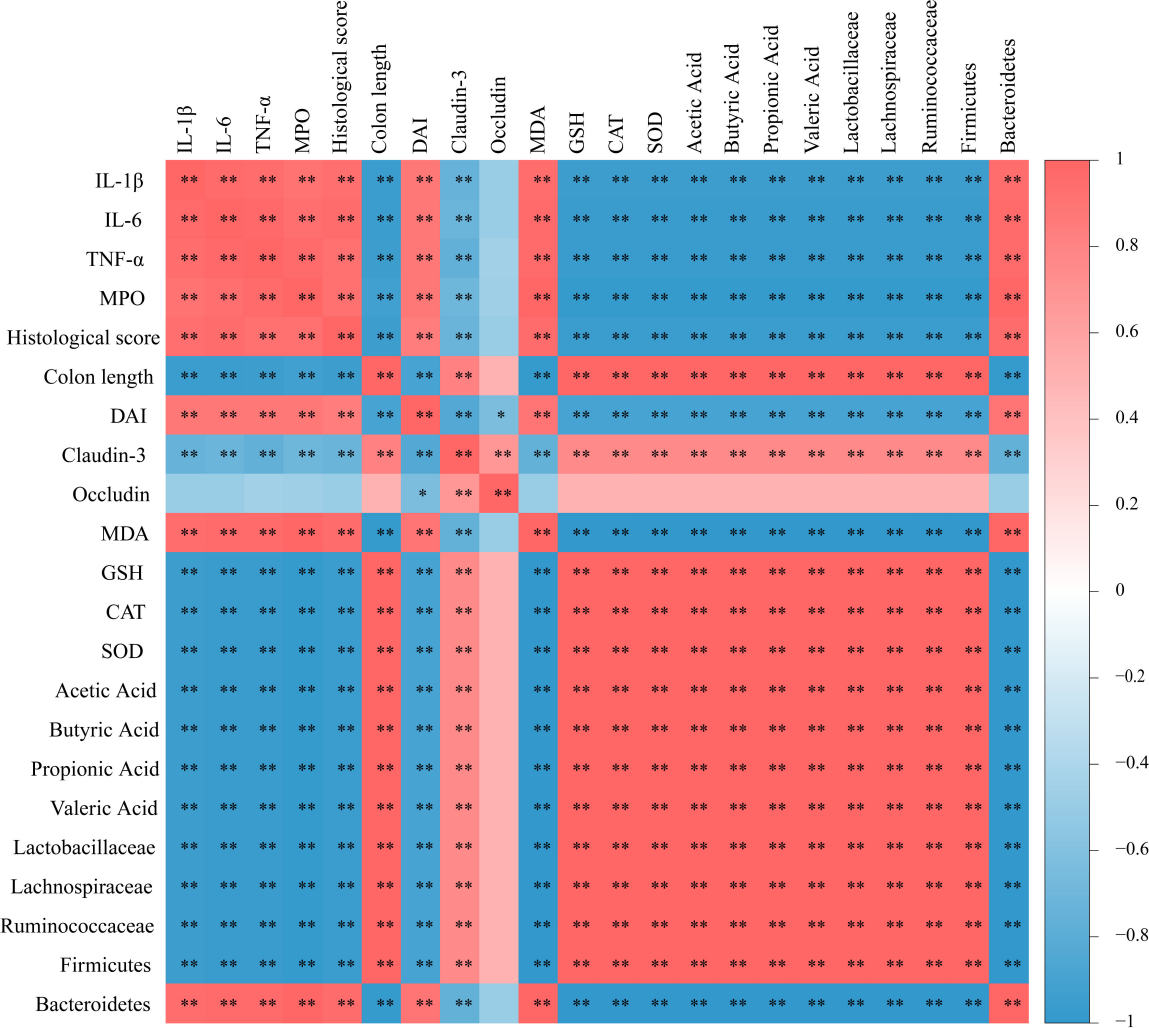
Effects of PHY on the relationship between microbiota and inflammatory markers, short-chain fatty acids, intestinal barrier, and oxidative stress. Assessment of the link between microbiota composition and inflammatory markers, intestinal barrier integrity, short-chain fatty acid levels, and oxidative stress parameters. Positive correlations are highlighted in red blocks, while negative correlations are depicted in blue blocks. The intensity of the color reflects the strength of the correlation. Each group consisted of five biological replicates (*n* = 5). *P < 0.05, **P < 0.01.

An analysis of the link between the relative abundance of gut microbiota and the levels of inflammatory factors, intestinal barrier proteins, and SCFAs was executed. The findings revealed a negative correlation between *Lactobacillaceae*, *Lachnospiraceae*, *Ruminococcaceae*, SCFAs, and intestinal barrier (Fig. 10). *Lactobacillaceae*, *Lachnospiraceae*, and *Ruminococcaceae* exhibited a negative association with inflammatory factors (Fig. 10). These statistical findings suggest a strong association between inflammatory factors, intestinal barrier proteins, SCFAs, and intestinal flora.

### PHY improves DSS-induced colitis in mice by regulating gut microbiota

**Fig 11:**
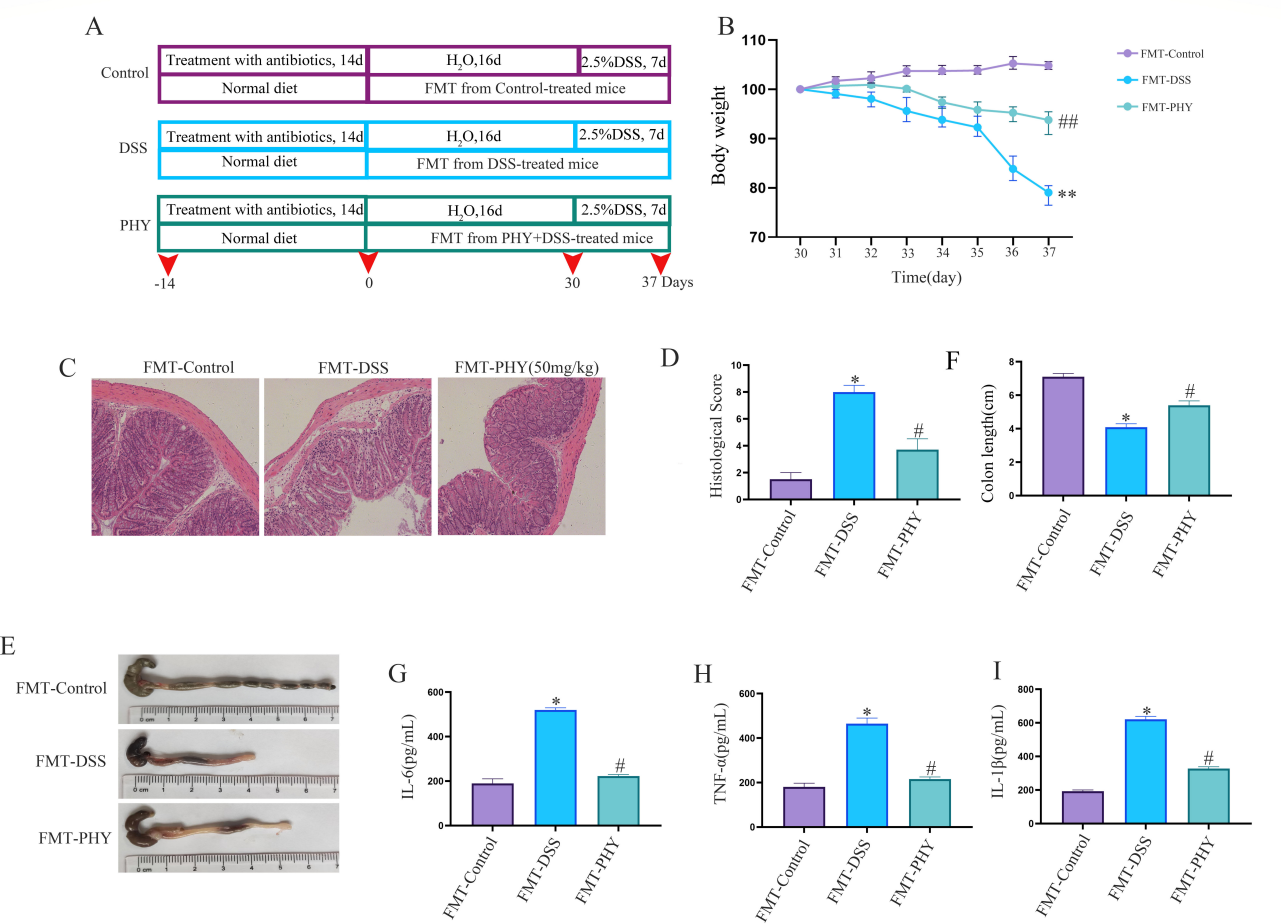
Effect of FMT on mice with DSS-induced colitis. (**A**) Experimental protocol. (**B**) Alterations in body weight among mice groups. (**C**) Histopathological examination using H&E staining in mouse groups; bar scale = 80 µm. (**D**) Histological scoring for individual mice groups. (**E**) Visual representation of colon length. (**F**) Comparative analysis of colon length across mice groups. Measurement of IL-6 (G), TNF-α (**H**), and IL-1β (**I**) levels via enzyme-linked immunosorbent assay. PHY concentration is 50 mg/kg. Data are depicted as the mean ± SEM. Each group consisted of six biological replicates (*n* = 6). *P < 0.05 compared to the control group; ^#^*P* < 0.05 compared to the DSS group.

An experimental design involving fecal bacteria transplantation was implemented to investigate whether PHY functions through the gut microbiota (Fig. 11A). The outcomes indicated that body weight (Fig. 11B) and colon length (Fig. 11E and F) were notably lowered in the DSS group in relation to the control group, and these trends were reversed after PHY pre-protection. H&E outcomes revealed severe crypt damage in the DSS group relative to the control group, whereas pathological damage improved following PHY pre-protection (Fig. 11C and D). Additionally, inflammatory factors were substantially enhanced in the DSS group in relation to the control group. Following FMT-PHY treatment, there was a considerable reduction in inflammatory factors (Fig. 11G through I). These outcomes reveal that PHY can alleviate DSS-induced colitis injury by modulating intestinal flora.

## DISCUSSION

**Fig 12:**
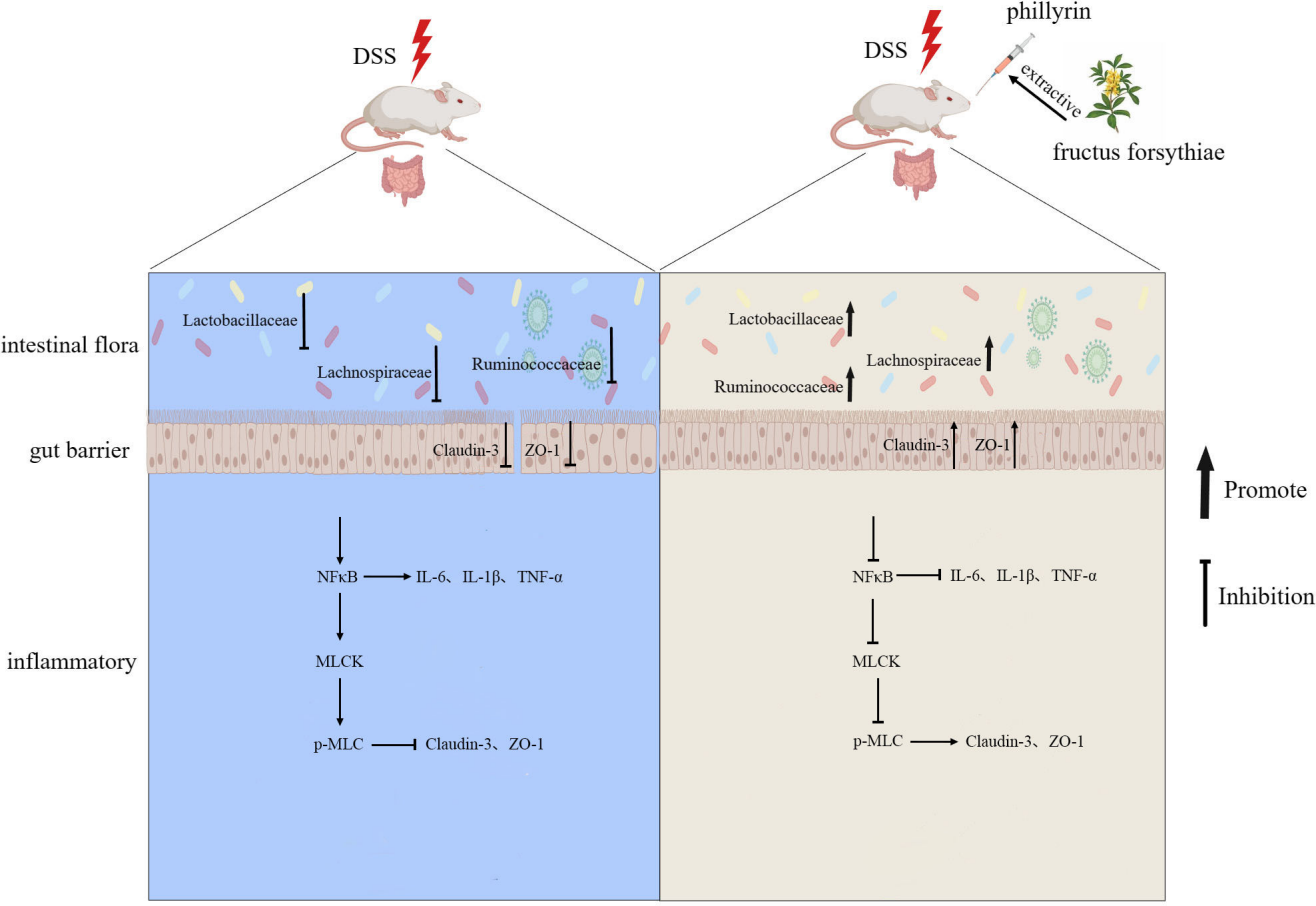
Table of Contents (TOC) graphic. Diagram summarizing our findings. Dietary PHY alleviated DSS-induced colitis by maintaining the intestinal barrier and regulating intestinal microbes.

Conventional treatment of IBD mainly includes anti-tumor necrosis factor drugs, 5-aminosalicylic acid, (5-ASA) and corticosteroids (24, 25). Among them, corticosteroids are one of the cornerstones of IBD drug treatment. A large number of studies have shown that the therapeutic effect of corticosteroids is better than 5-ASA (26, 27). However, long-term use of glucocorticoids can also lead to many adverse reactions and complications, such as osteoporosis (27–29). Therefore, reducing the dose of corticosteroids as much as possible in combination with other medications to maintain remission is the treatment of choice. At present, the application of plant metabolites in the treatment of enteritis has become one of the current research hotspots (12, 30). PHY has anti-inflammatory (31), anti-viral, and immunomodulatory effects (32). Despite its known pharmacological effects, there are limited reports on the effect of PHY on DSS-induced colitis. The current investigation confirmed that PHY mitigates DSS-induced colitis by modulating intestinal flora and repairing the intestinal barrier (Fig. 12).

UC is characterized by chronic, non-specific colon and rectum inflammation (33). Its primary clinical manifestations include diarrhea, rectal bleeding, weight loss, abdominal pain, and the presence of blood in stool mucus (34). The findings of the present investigation demonstrated that PHY ameliorated colitis symptoms comprising hematochezia, weight loss, diarrhea, spleen enlargement, and colon shortening in colitis mice. The NF-κB pathway is pivotal in inflammatory response (35). The present investigation observed notably elevated pro-inflammatory cytokine levels (TNF-α, IL-6, and IL-1β) in the colon of mice with DSS-induced colitis. PHY notably inhibits pro-inflammatory factor secretion by reducing the phosphorylated NF-κB expression. Moreover, oxidative stress is recognized as one of the potential pathophysiological mechanisms of IBD (36). Excessive oxygen free radicals are pivotal in the development of colitis, leading to ulcerative inflammatory tissue damage. Therefore, inhibiting intestinal oxidative stress may be an effective strategy to treat colitis. In this research, PHY considerably increased the activities of SOD, CAT, and total antioxidant capacity in DSS-induced mouse tissues, reduced the content of MDA, and enhanced the antioxidant capacity.

The integrity of intestinal barrier function is intricately linked to the inflammatory response of intestinal mucosa and the production of various inflammatory cytokines (37). The integrity of intestinal barrier function is intricately linked to the inflammatory response of intestinal mucosa and the production of various inflammatory cytokines (38). Activated NF-κB can bind to the MLCK promoter, thereby enhancing MLCK (39, 40). Consistent with present outcomes, MLC and NF-κB phosphorylation levels were considerably enhanced in the DSS group but substantially reduced after PHY pre-protection.

SCFAs can regulate intestinal cell energy metabolism, protect the intestinal mucosal barrier, and inhibit inflammatory response and cell proliferation and differentiation (9, 41). One of the investigations revealed that butyric acid and acetic acid in the stool of individuals with UC were notably reduced in comparison to healthy controls (42). Consistent with the outcomes of the present research, PHY pre-protection can mitigate the reduction of SCFAs induced by DSS.

Gut microbiota and its derived metabolites are pivotal in the pathogenesis of UC, serving as critical mediators in the interaction between the host and gut microbiota (43). The *Firmicutes*:*Bacteroidetes* ratio (F:B) is widely considered crucial for maintaining intestinal homeostasis, with a lower ratio typically associated with the onset of IBD. In alignment with the present research outcomes, the results illustrate that PHY pre-protection substantially enhances the F:B ratio reduction induced by DSS (44). Functioning as probiotics, *Lactobacillaceae* and *Lachnospiraceae* can secrete SCFAs to protect intestinal mucosa and attenuate inflammation (45–47). The findings depict that PHY treatment can substantially enhance *Lactobacillaceae* and *Lachnospiraceae*. Moreover, a proportional relationship exists between *Lactobacillaceae* and *Lachnospiraceae* with SCFAs, while an inverse correlation is observed with inflammatory factors and DAI. *Ruminococcaceae*, a bacterium known for producing butyrate, may ameliorate colitis by reducing intestinal cellular injury and suppressing the production of pro-inflammatory cytokines in the colon (48). Consistent with our study, PHY treatment can significantly increase *Ruminococcaceae* and is directly proportional to SCFAs and oxidative stress and inversely proportional to DAI.

Furthermore, FMT was utilized to confirm whether PHY-induced alterations in gut microbiota are crucial in DSS-induced protection in mice. The outcomes revealed that PHY-induced microbiota transplantation resulted in weight gain and elongation of colon length in comparison to the DSS group. Based on histopathological evaluation, the FMT group exhibited reduced crypt destruction and ulcer formation. Additionally, IL-1β and IL-6 levels in the colon were decreased in the FMT group, and intestinal barrier integrity was repaired. Consequently, PHY ameliorates colitis induced by DSS by modulating the structure of gut microbiota. However, our current study is limited to animal models, and more human clinical trials are needed in the future to demonstrate the effectiveness of the microbiome.

In summary, PHY can mitigate colitis induced by DSS by preserving the intestinal barrier, balancing the redox state, and regulating intestinal flora and SCFA levels. These findings provide a solid scientific foundation for potentially developing functional foods utilizing PHY for therapeutic purposes. However, before future clinical use, we need to further explore the safety and efficacy of PHY in humans, as well as explore its pharmacokinetics and long-term effects.

## Data Availability

Bacterial flora data of fecal microbiota transplantation have been uploaded to National Center for Biotechnology Information Public database Sequence Read Archive under accession number PRJNA1184361.
